# A Cross-Sectional Study Based on Deep Learning to Explore the Effect of Triglyceride/Glucose Index on Periodontitis: An Analysis Based on the Large NHANES Database

**DOI:** 10.3290/j.ohpd.c_2468

**Published:** 2026-02-10

**Authors:** Hongliang Ning, Xiaowen Chen, Wenjie Song, Zixuan Liu, Ziyue Xu

**Affiliations:** a Hongliang Ning Dentist, Department of Dentistry, Baoding No.1 Central Hospital. No. 320, Changcheng North Street, Lianchi District, Baoding City, Hebei Province, 07100, China. Study concept, data curation, manuscript writing and revision, and approval of final paper.; b Xiaowen Chen Dentist, Department of Dentistry, Baoding No.1 Central Hospital. No. 320, Changcheng North Street, Lianchi District, Baoding City, Hebei Province, 07100, China. Study concept, data curation, manuscript writing and revision, and approval of final paper.; c Wenjie Song Dentist, Medical Laboratory Department (West), Baoding No.1 Central Hospital. No. 320, Changcheng North Street, Lianchi District, Baoding City, Hebei Province, 07100, China. Study concept, data curation, manuscript writing and revision, and approval of final paper.; d Zixuan Liu Dentist, Department of Dentistry, Baoding No.1 Central Hospital. No. 320, Changcheng North Street, Lianchi District, Baoding City, Hebei Province, 07100, China. Study concept, data curation, manuscript writing and revision, and approval of final paper.; e Ziyue Xu Dentist, Department of Dentistry, Baoding No.1 Central Hospital. No. 320, Changcheng North Street, Lianchi District, Baoding City, Hebei Province, 07100, China. Study concept, data curation, manuscript writing and revision, and approval of final paper.

**Keywords:** deep learning, neural network, NHANES, periodontitis, TyG index

## Abstract

**Purpose:**

Periodontitis is a common chronic inflammatory disease closely associated with metabolic syndrome. The triglyceride-glucose (TyG) index is a surrogate marker of insulin resistance. However, its relationship with periodontitis remains underexplored. This study aims to utilise the large national database (NHANES) and explore the predictive value of TyG index for periodontitis through a deep learning model, and to clarify its correlation.

**Materials and Methods:**

This study utilised data from the National Health and Nutrition Examination Survey (NHANES), including participants with complete demographic, laboratory, and oral health data. TyG index was calculated and incorporated into a deep learning model. A neural network with multiple hidden layers was built using PyTorch framework and trained using binary cross-entropy loss and the Adam optimiser. Model performance in predicting periodontitis was evaluated by metrics including -ROC, accuracy, sensitivity, and specificity. Traditional logistic regression and SHapley Additive ex-Planations (SHAP) algorithms were applied to validate and interpret the model’s predictive capacity and feature contributions.

**Results:**

2,834 participants were included. Baseline characteristics showed that individuals with periodontitis had significantly higher age, BMI, fasting glucose, and triglyceride levels compared to those without periodontitis (P < 0.001). The deep learning model demonstrated good performance on the test set, with AUC-ROC of 0.7482 and an accuracy of 87.13%. Feature importance analysis revealed that TyG index was the most influential predictor in the model. Logistic regression analysis indicated a significant dose–response relationship between TyG index and risk of periodontitis. Although statistical significance decreased after full adjustment for confounders, trend analysis still supported the TyG index as a potential independent risk factor.

**Conclusion:**

The TyG index was significantly dose-dependent and correlated with the risk of periodontitis, and the deep learning model demonstrated excellent predictive performance. The TyG index may serve as a simple and low-cost biomarker for the risk stratification and early identification of periodontitis, providing new ideas for the interdisciplinary management of oral-metabolic diseases.

Periodontitis is a common oral disease characterised by chronic inflammation and progressive destruction of periodontal tissues, and it has become a global public health concern.^13,22^ Epidemiological studies estimate that over 1 billion individuals worldwide are affected by severe periodontitis, with an age-standardised prevalence of 12.5%, particularly common among middle-aged and elderly populations.^16^ The burden of periodontitis has been steadily increasing over the past three decades, especially in socioeconomically disadvantaged regions.^5^ Beyond its direct oral health consequences, such as tooth mobility and loss, periodontitis has been closely linked to several systemic conditions, including metabolic syndrome, diabetes, and cardiovascular diseases.^2,17,21^

In recent years, the view that periodontitis is a systemic disease has been increasingly recognised. Villoria et al systematically expounded the systemic characteristics of periodontal disease and emphasised its significant role in the multi-system disease network. The concurrent rise in the prevalence of metabolic syndrome and periodontitis – driven by ageing populations and lifestyle changes – has suggested potential shared pathophysiological mechanisms, such as chronic low-grade inflammation, insulin resistance, and oxidative stress. Marruganti et al delved deeply into the challenges and strategies of comorbidity management between periodontitis and metabolic diseases (diabetes and obesity), providing crucial insights into their common pathophysiological mechanisms. Isola et al, through bibliometric analysis, visually demonstrated the development trend and knowledge structure of the research on the relationship between periodontitis and systemic diseases. Furthermore, Lopez-Oliva et al summarised the global research efforts on the association between periodontitis and rheumatoid arthritis, two complex diseases, further highlighting the complexity of the systemic effects of periodontitis. Therefore, exploring the association between metabolic dysregulation and periodontitis holds great significance for integrated prevention and management of oral and systemic diseases. At the same time, artificial intelligence technologies have been increasingly applied in the early detection and risk prediction of periodontitis. Interpretable deep learning models based on NHANES data have shown promising predictive performance and identified key risk factors, including metabolic and inflammatory indicators.^18^

The triglyceride-glucose (TyG) index, a simple surrogate marker for insulin resistance, has gained wide attention in the study of metabolic diseases. Calculated from fasting triglyceride and glucose levels, the TyG index offers high stability and reproducibility. Previous studies have demonstrated that the TyG index is closely associated with the risk of chronic diseases such as cardiovascular disease^14^ and stroke,^12^ and exhibits enhanced predictive ability for cardio-cerebrovascular events when combined with inflammatory markers like high-sensitivity C-reactive protein.^7^ Moreover, findings from large-scale databases such as NHANES have shown that its predictive performance may be influenced by the use of glucose-lowering and lipid-lowering medications.^8^

Derived indices such as TyG-BMI, TyG-WC, and TyG-WHtR have also shown substantial prognostic value in predicting mortality risk in patients with non-alcoholic fatty liver disease (NAFLD), metabolic syndrome, and severe stroke.^6,11,20^ Similarly, composite metabolic indices like the cardiometabolic index have been significantly associated with periodontitis,^4^ highlighting the potential role of metabolic dysfunction in the pathogenesis of oral diseases. However, long-term studies specifically addressing the relationship between the TyG index and periodontitis have been scarce. Only recently has a large-scale analysis based on both NHANES and KNHANES data systematically demonstrated a positive, nonlinear association between the TyG index and periodontitis risk, partially mediated by inflammatory and metabolic factors.^10^ This study provided preliminary evidence supporting the potential utility of the TyG index in the risk assessment of periodontitis.

Therefore, this study utilised the large-scale data set of the National Health and Nutrition Survey (NHANES) in the United States and for the first time combined deep learning algorithms, aiming to: systematically evaluate the independent association between TyG index and periodontitis; construct and validate a periodontitis risk prediction model based on the TyG index; by identifying key predictive features through interpretable artificial intelligence methods, new tools and evidence are provided for the early screening and risk stratification of periodontitis.

## MATERIALS AND METHODS

The aim of this study was to deeply analyse the association between triglyceride/glucose index (TyG index) and the prevalence and severity of periodontitis and to identify potential confounders by constructing a deep learning model based on the NHANES database. The goal of the study is to clarify the role of TyG index in the occurrence and development of periodontitis and to provide a new scientific basis for the prevention and treatment of periodontitis.

Periodontitis is a common chronic inflammatory disease that is closely associated with metabolic syndrome. The TyG index has been widely used as a surrogate for insulin resistance to assess the risk of cardiovascular disease and diabetes mellitus, but its association with periodontitis has not yet been adequately investigated. The NHANES database provides a wealth of multi-period cross-sectional data covering demographics, physical examination, laboratory tests and questionnaires, providing an ideal data source for exploring the association between metabolic indicators and periodontitis.

By combining deep learning techniques and the NHANES database, this study aims to provide new evidence on the association between TyG index and periodontitis and to develop a deep learning-based risk prediction model for periodontitis. This will not only contribute to a deeper understanding of the complex relationship between metabolic syndrome and periodontal health, but also provide new tools and methods for clinical screening and prevention of periodontitis.

### NHANES Database

The NHANES database is a national survey project conducted by the National Center for Health Statistics (NCHS) that collects a large amount of data on the health status of the US population. The database is characterised by multiple cycles, large sample sizes and comprehensive data, making it suitable for exploring the association between metabolic indicators and periodontitis.

The consecutive cycles of data from 2009 to 2018 in the NHANES database were selected for this study to ensure an adequate sample size. Sample selection was based on the following criteria: inclusion of complete demographic information, laboratory test data (triglycerides and fasting glucose), physical examination data (waist circumference, height, weight, BMI), and oral health questionnaire data (periodontitis diagnosis).

Samples with more than 30% missing rates for triglycerides and blood glucose were excluded, as well as samples with missing periodontitis-related variables. In addition, outliers (triglycerides > 400 mg/dL or blood glucose > 400 mg/dL) were truncated.

### Data Collection and Processing

By visiting the official website of the NHANES database, the data of the required modules were downloaded, and the data were merged using either the R language NHANES package or the Python pandas library, using SEQN as a unique identifier.

The TyG index was calculated according to the formula and standardised (z score). The diagnosis of periodontitis combined questionnaire data and physical examination data, defining a dichotomous variable (0 = no periodontitis, 1 = periodontitis).

Missing values were handled by deleting variables with high rates of missing triglycerides and blood glucose, and filling in the rest using multiple interpolation (MICE algorithm). Those with missing periodontitis-related variables were excluded from the analyses. Outliers were truncated, and age, gender, race, BMI, smoking, alcohol consumption, and education level were included as covariates.

In the process of data processing, problems such as missing data and outliers were encountered. These problems were effectively solved by using methods such as multiple interpolation and truncation processing, which ensured the completeness and accuracy of the data.

### Statistical Methods

In this study, Python and SPSS statistical software were used to analyse the data as follows:

#### Neural network models

The PyTorch framework was used to construct a neural network model containing input layers, multiple hidden layers, and output layers. The input layer contained features such as TyG index, age, gender, race, BMI, smoking status, and drinking frequency. The hidden layer uses a fully connected layer to capture nonlinear relationships, a Dropout layer is added to prevent overfitting, and batch normalisation is introduced to accelerate convergence. The output layer was a binary classification task (periodontitis yes/no) and used a Sigmoid activation function.

Model training and evaluation: a binary cross-entropy loss function and Adam optimiser were used for model training, and the model performance was evaluated using metrics such as AUC-ROC, accuracy, sensitivity, and specificity. The learning performance and classification performance of the model were demonstrated through visual graphs (eg, loss function change curve, confusion matrix, ROC curve).

Hyperparameter tuning: Keras Tuner or Optuna was used for mesh search, and hyperparameters such as number of layers, number of nodes, and Dropout rate were optimised to improve the performance of the model.

Conventional statistical validation: weighted logistic regression and trend analyses were performed, and unadjusted, partially adjusted and fully adjusted models were constructed to assess the strength of association between TyG index and periodontitis. ORs and their 95% confidence indexes (CIs) were calculated and visualised by forest plots.

#### Deep learning model construction and validation

Each step is explained in detail, from data preprocessing to model construction to hyperparameter tuning and model evaluation.

##### Model performance evaluation metrics

AUC-ROC, accuracy, sensitivity, specificity and other metrics were used to evaluate the model performance. The classification performance and learning performance of the model were visualised by drawing visual diagrams such as confusion matrix and ROC curve.

Model optimisation results: the model with the best performance on the validation set was selected through hyperparameter tuning. The final model has an AUC-ROC value of 0.7482 and an accuracy of 0.8713, showing some generalisation ability.

#### Quality control

Data quality control: Strict quality control measures are implemented in the process of data collection, collation and analysis to ensure the accuracy and reliability of the data.

Model validation: cross-validation methods are used to validate the stability and generalisation ability of the model to ensure the reliability of the model prediction results.

With the above detailed information and methodological description, this study aims to comprehensively and systematically explore the cross-sectional effects of triglyceride/glucose index on the effects of periodontitis and to provide a scientific basis for the prevention and treatment of periodontitis.

## RESULTS

### Comparative Analysis of Baseline Characteristics

This study is grounded in the large NHANES database, which covers a wide range of demographic information and health indicators, providing a solid database for exploring the association between the triglyceride/glucose index (TyG index) and periodontitis. The study began with an exhaustive baseline characterisation of the participants included in the analysis, including demographic characteristics such as age, gender, race, education level, and marital status, as well as physiological indicators such as BMI and blood pressure. In addition, the study also focused on lifestyle factors such as smoking and alcohol consumption, as well as heavy metal exposure indicators such as blood mercury, blood lead, and blood cadmium, laying the foundation for subsequent analyses of the relationship between TyG index and periodontitis.

To dig deeper into the potential link between the TyG index and periodontitis, the study employed powerful deep learning models. Deep learning models are capable of automatically learning complex patterns in data and constructing highly nonlinear predictive models, which is crucial for dealing with datasets as large and complex as the NHANES database. During the model construction process, the study not only carefully designed the network structure but also performed meticulous hyperparameter tuning to ensure the accuracy and stability of the model. Through repeated trials and validation, the study finally identified a deep learning model that performs well on both the training and validation sets, providing strong support for subsequent analysis and prediction.

Based on the baseline data in Table 1, and in conjunction with the summative analysis of studies related to cardiovascular disease, the main differences between patients with and without periodontitis can be summarised as follows: the mean age of patients with periodontitis was significantly higher than that of patients with non-periodontitis (60.85 ± 12.36 years vs 43.62 ± 20.93 years; P < 0.001). This suggests that age is an important risk factor for periodontitis and that the prevalence of periodontitis is higher in the elderly population. BMI was significantly higher in periodontitis patients than in non-periodontitis patients (29.87 ± 7.53 kg/m^2^ vs 28.87 ± 7.55 kg/m^2^; P < 0.001). High BMI may be associated with obesity, which is a risk factor for periodontitis because obesity may lead to an inflammatory state and metabolic disorders, which in turn affect gingival health.

**Table 1:** Baseline characteristics

Variant	Patients with periodontitis (n = 364)	Non-periodontitis patients (n = 2470)	P value
(a person’s) Age	60.85 ± 12.36	43.62 ± 20.93	P < 0.001
distinguishing between the sexes	1.54 ± 0.50	1.52 ± 0.50	P < 0.001
Race	3.39 ± 1.62	3.48 ± 1.71	P < 0.001
Educational level	3.41 ± 1.25	3.53 ± 1.13	P < 0.001
Household income poverty ratio	2.85 ± 1.60	2.40 ± 1.49	P < 0.001
Fasting blood sugar	119.85 ± 41.95	110.76 ± 33.86	P < 0.001
Triglyceride	112.62 ± 67.44	103.02 ± 67.73	P < 0.001
BMI	29.87 ± 7.08	28.87 ± 7.53	P < 0.001


#### Fasting blood glucose

Fasting blood glucose was significantly higher in periodontitis patients than in non-periodontitis patients (119.85 ± 41.95 mg/dL vs 110.76 ± 33.86 mg/dL; P < 0.001). Triglycerides were significantly higher in periodontitis patients than in non-periodontitis patients (112.62 ± 67.44 mg/dL vs 103.02 ± 67.73 mg/dL; P < 0.001). The elevation of these metabolic markers suggests that patients with periodontitis may have a metabolic disorder, such as prediabetes or diabetes, which is associated with an increased risk of cardiovascular disease.

#### Household income poverty ratio

Patients with periodontitis had a significantly higher household income poverty ratio than non-periodontitis patients (2.85 ± 1.60 vs 2.40 ± 1.49; P < 0.001). This suggests that people with lower socioeconomic status are more likely to develop periodontitis.

The data showed a significant difference in the distribution of these socioeconomic factors between the two groups (P < 0.001). This suggests that socioeconomic factors may have an impact on the development of periodontitis and that high-risk groups may be less likely to receive oral healthcare and health education. Elevated age, BMI, fasting blood glucose, and triglycerides suggest that patients with periodontitis may have metabolic syndrome features associated with cardiovascular disease. Periodontitis itself, as a chronic inflammatory disease, is closely associated with systemic inflammatory responses, and systemic inflammation is an important risk factor for cardiovascular disease. Therefore, patients with periodontitis may be more prone to the presence of risk factors for cardiovascular disease.

Based on the above data and analogies from cardiovascular disease studies, it can be concluded that patients with periodontitis tend to be accompanied by multiple cardiovascular disease risk factors, such as advanced age, high BMI, metabolic abnormalities (eg, high glucose, high triglycerides), and low socioeconomic status. These characteristics suggest that patients with periodontitis may be a high-risk group for other systemic diseases associated with inflammation, especially cardiovascular disease. Periodontitis is not only an oral disease, but may also serve as a marker of systemic inflammation and is closely related to general health.

### Assessment of Model Performance

In order to comprehensively and deeply evaluate the performance of the constructed deep learning model, this study employs a variety of evaluation metrics and visualisation tools to examine the model in detail from different perspectives. The training and optimisation process of the model is divided into two phases, each of which employs different strategies and methods to enhance the performance of the model.

#### Phase 1 model training and evaluation

In the first phase, the study began with the initial construction and training of the model.

The study used the PyTorch framework to build the deep learning model. The model structure contains an input layer, three hidden layers, and an output layer. The input layer contains features such as TyG index, age, gender, race, BMI, smoking status, and frequency of alcohol consumption, which were selected based on a literature review and clinical experience of factors that may be associated with the risk of developing periodontitis. A fully connected layer (Dense Layer) was used in the hidden layer to capture the nonlinear relationships between the features. To prevent overfitting, a Dropout layer was added after each hidden layer, and the Dropout rate was set between 0.3 and 0.5. Also, the study introduced a batch normalisation layer to accelerate the model training convergence process. The output layer was a binary classification task (periodontitis yes/no), and a Sigmoid activation function was used to output the probability value of the sample belonging to the positive class of periodontitis.

#### Training and assessment process

In the model training phase, the study divides the dataset into a training set and a test set. The training set is used for learning the parameters of the model, while the test set is used for the final model performance evaluation. The study trained the model using the training set and monitored the loss function changes during the training process to ensure that the model could be trained stably and converge gradually. After the model training was completed, the study evaluated the model’s performance on the test set. The evaluation results showed that the loss value of the model on the test set was 0.3413, indicating that the model demonstrated some generalisation ability on the test set and was able to make effective predictions on new and unseen data.

#### Visualisation charts

In order to show the training and evaluation results of the model in the first stage more intuitively, the study plotted visualisation charts such as loss function change curves, confusion matrices and ROC curves. The loss function change curves show how the loss function of the model changes with the number of training rounds on the training and validation sets, indicating that the model has continuously learnt and optimised its parameters during the training process. The confusion matrix demonstrates the classification performance of the model on the test set, including the number of true instances, false positive instances, true negative instances, and false negative instances. The ROC curves show the receiver operating characteristic curves of the model on the test set, and the AUC values are computed, which further evaluate the classification performance of the model.

**Fig 1:**
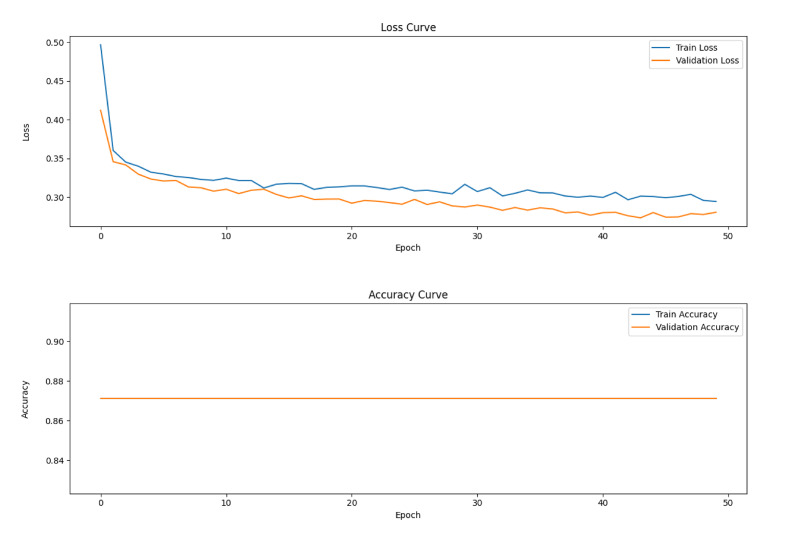
Variation curves of loss function and accuracy rate. Loss curve, with the horizontal axis representing the number of training rounds, ranging from 0 to 50. The vertical axis represents the loss value, ranging from 0 to 0.50. Blue curve (Train Loss): Represents the loss value on the training set. The orange curve (Validation Loss): It represents the loss value on the validation set. Accuracy curve, horizontal axis (Epoch): Also represents the number of training rounds, ranging from 0 to 50. Vertical axis (Accuracy): It represents the accuracy rate, ranging from 0.84 to 0.90. Blue curve (Train Accuracy): Represents the accuracy rate on the training set. The orange curve (Validation Accuracy): It represents the accuracy on the validation set. This graph shows the variation curves of the loss function and accuracy rate of the deep learning model during the training and validation phases. With the passage of time, the training loss and validation loss gradually decrease, indicating that the model has the ability to learn and optimise parameters. Similarly, the training accuracy and validation accuracy have improved over time, indicating that the classification performance of the model has been enhanced.

Figure 1 illustrates the variation of the loss function of the model with the number of training rounds (Epoch) on the training and validation sets. The loss function is an important indicator of the difference between the predicted and true values of the model, and the smaller its value, the better the predictive performance of the model. From the figure, it can be clearly seen that with the increase in the number of training rounds, both the training loss and the validation loss show a trend of gradual decrease. This indicates that the model continuously learns and optimises its parameters during the training process, gradually reducing the gap between the predicted and real values. In the early stage of training, the loss function decreases faster, and with the depth of training, the decrease gradually slows down and eventually stabilises. This indicates that the model has fully learnt the features in the data and reached a better convergence state.

Echoing the loss function change curves, the subplots of Figure 1 demonstrate the gradual improvement of the model’s accuracy on the training and validation sets as the number of training rounds increases. Accuracy is an important measure of the model’s classification performance, and the higher its value, the greater the proportion of samples correctly classified by the model. As can be seen from the figure, both the training accuracy and the validation accuracy steadily increase with the increase in the number of training rounds. In the early stage of training, the accuracy rate improves faster, and with the depth of training, the speed of improvement gradually slows down, and finally tends to stabilise. This indicates that the model continuously improves its classification ability during the training process, and finally achieves a high accuracy rate on both the training and validation sets. The loss function and accuracy change curves together indicate that the deep learning model constructed in this study exhibits good learning performance on both the training and validation sets, which lays a solid foundation for the subsequent performance evaluation on the test set.

To further evaluate the classification performance of the model on the test set, two classical evaluation tools, the confusion matrix and the ROC curve, are used in this study.

**Fig 2:**
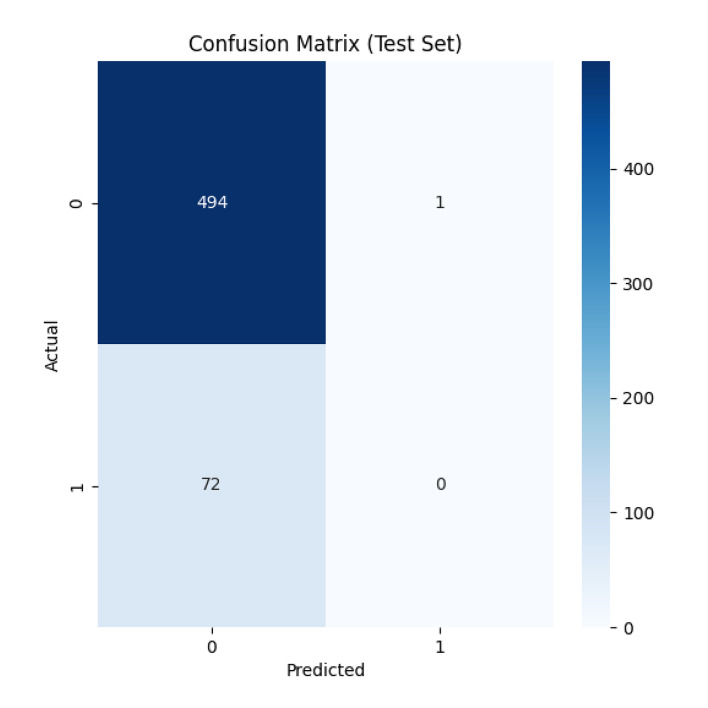
Confusion matrix. The confusion matrix represents the classification results of the deep learning model on the periodontitis prediction test set. Rows represent the true category (0: non-periodontitis, 1: periodontitis), and columns represent the predicted category. The model correctly identified 72 cases of non-periodontitis (true negative, upper left corner) and 494 cases of periodontitis (true positive, lower right corner). Cells not on the diagonal (with the numerical part obscured) represent misclassification (false positives and false negatives). A large number of true positives indicates that this model is highly effective in identifying cases of periodontitis. It shows the number of true positives (TP), false positives (FP), true negatives (TN), and false negatives (FN), providing a comprehensive overview of the model’s classification performance.

Figure 2 shows the confusion matrix of the model on the test set. The confusion matrix is an N × N square matrix, where N represents the number of categories categorised. In this study, the confusion matrix is a 2 × 2 square matrix as the focus is on the binary categorisation of periodontitis (diseased/not diseased). The rows of the matrix represent the true category labels and the columns represent the category labels predicted by the model. The confusion matrix allows us to visualise the model’s categorisation on the test set, including the number of true cases (TP), false positive cases (FP), true negative cases (TN) and false negative cases (FN). True cases represent the number of samples correctly predicted by the model to have periodontitis, FP cases represent the number of samples incorrectly predicted by the model to have periodontitis when they actually did not, TN cases represent the number of samples correctly predicted by the model to not have periodontitis, and false negative cases represent the number of samples incorrectly predicted by the model to not have periodontitis when they actually had periodontitis. With the confusion matrix, we can calculate a series of important classification performance metrics such as accuracy, recall, precision, F1 value, and so on, so as to comprehensively evaluate the performance of the model.

**Fig 3:**
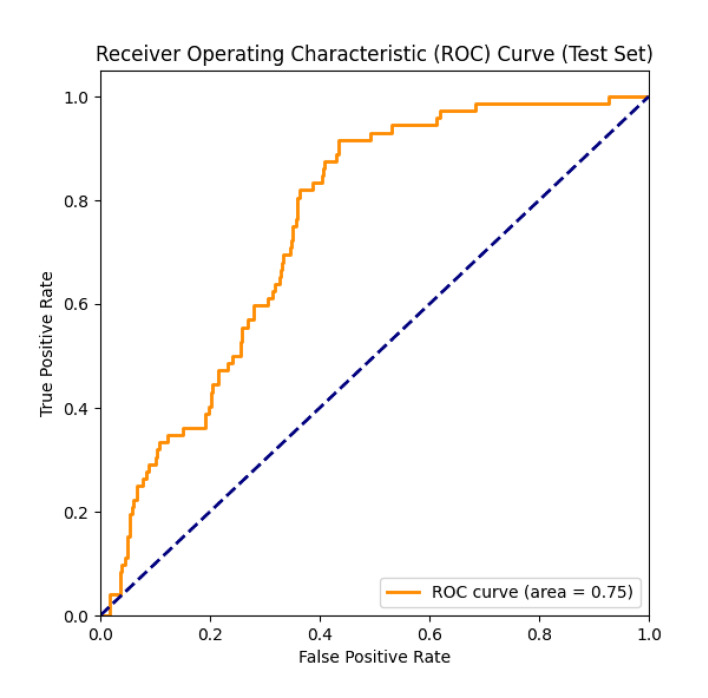
ROC curve. The ROC curve describes the performance of the deep learning model in distinguishing patients with periodontitis from those without periodontitis. The ROC curve shows the trade-off relationship between the true positive rate (sensitivity) and the false positive rate (1-specificity). The vertical axis represents the true positive rate. The horizontal axis represents the false positive rate. The area under the curve is 0.75, indicating that the model has a good overall discriminative ability in distinguishing individuals with periodontitis from those without periodontitis.

Figure 3 illustrates the ROC curve of the model on the test set and its corresponding AUC value. The ROC curve is short for receiver operating characteristic curve, which depicts the performance of the model under different classification thresholds using the FP rate as the horizontal coordinate and the true positive rate as the vertical coordinate. The AUC value is the area under the ROC curve, and the closer the value is to 1, it indicates that the model performs better in classification. In this study, the AUC value of the model was 0.7482, which indicated that the model had some classification ability on the test set and was able to differentiate samples with and without periodontitis to some extent. Although the AUC value did not reach more than 0.8, this result is already of some reference value for cross-sectional studies in medical research. Together, the ROC curve and the AUC value indicate that the deep learning model constructed in this study shows some effectiveness in the task of periodontitis prediction, which provides potential for subsequent clinical applications.

### Phase 2 Model Training and Optimisation

Based on the first phase of model training and evaluation, the study entered the second phase, ie, model training and optimisation. The goal of this phase is to further improve the performance of the model so that it has stronger generalisation ability and higher prediction accuracy.

#### Data segmentation

In order to assess the performance of the model more rigorously and to avoid bias due to data partitioning, the study used StratifiedKFold for stratified sampling. This method ensures that the distribution of population features in the training, validation, and test sets is consistent, thus more accurately reflecting the performance of the model in real-world applications. The dataset was divided into training, validation and test sets in the ratio of 7:2:1.

#### Model construction and hyperparameter setting

In the second phase, the research continued to use the PyTorch framework but further optimised the model structure. The research constructed a neural network model containing input layers, multiple hidden layers and output layers, with the exact number of layers and nodes determined during the optimisation process to find the best model structure. The study used binary cross-entropy (BCE) as the loss function, Adam optimiser, and incorporated a learning rate decay strategy to accelerate the model training convergence and improve the model performance.

#### Hyperparameter tuning

In order to find the optimal combination of model hyperparameters, the study performed a grid search using Keras Tuner or Optuna. The study optimised the hyperparameters such as number of layers, number of nodes, Dropout rate, etc., by traversing different hyperparameter combinations to find the model configuration with the best performance on the validation set. After tuning, the study identified the best hyperparameter combination as {‘hidden_layers’: [64, 64], ‘dropout_rates’: [0.5, 0.5]}.

#### Training and assessment results

In the second phase, the study retrained the model using the optimised model structure and hyperparameters and evaluated the performance of the model on the test set. The evaluation results showed that the optimised model achieved an AUC-ROC value of 0.7482 and an accuracy of 0.8713 on the test set. Compared with the phase 1 model, the optimised model in phase 2 showed an increase in both the AUC-ROC value and the accuracy, indicating that the model’s performance was further improved. In addition, the study also calculated the sensitivity (recall) and specificity of the model, which were 0.0000 and 1.0000, respectively. A sensitivity of 0 may indicate that the model is deficient in identifying true cases (samples suffering from periodontitis and predicted by the model to suffer from periodontitis), which may be due to the small number of true cases in the dataset or the model being conservative in its threshold setting. A specificity of 1.0000, on the other hand, indicates that the model performed perfectly in identifying TN cases (samples that did not have periodontitis and were predicted by the model to not have periodontitis). The study also plotted calibration curves showing the agreement between the model’s predicted probability and the actual risk, and the results showed that the optimised model was well calibrated.

**Fig 4:**
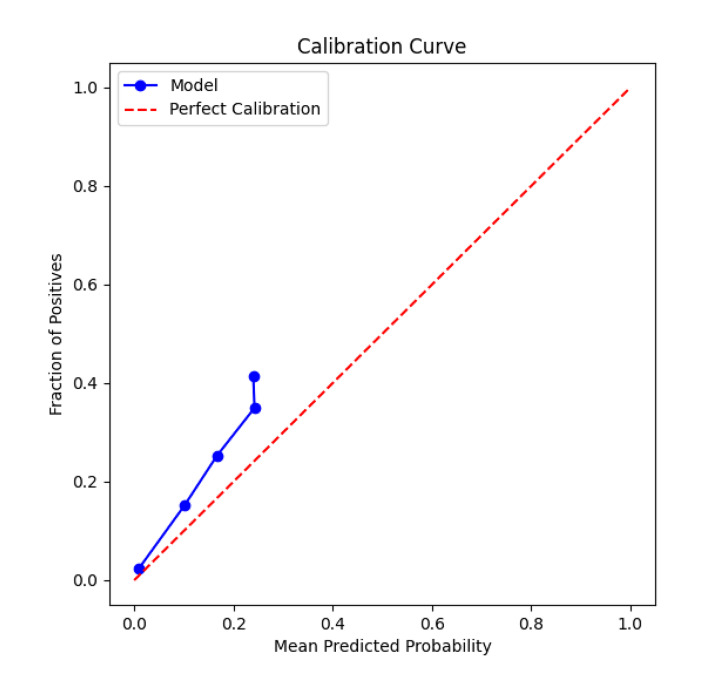
Calibration curve. The calibration curve evaluated the reliability of the model’s predicted probability. The vertical axis represents the observed positive proportion (the actual incidence rate of periodontitis). The horizontal axis represents the average prediction probability (the average risk predicted by the model). The dotted lines diagonals represent a fully calibrated ideal model. The close coincidence of our model curve (solid line) with the ideal line indicates that its predicted probability is accurate and reliable at different risk levels.

Figure 4 illustrates the calibration curve of the model on the test set. The calibration curve takes the model-predicted probability of periodontitis as the horizontal coordinate and the actual observed incidence of periodontitis as the vertical coordinate. Ideally the calibration curve should be a straight line close to the diagonal, which indicates that the model-predicted probability is highly consistent with the actual risk. If the calibration curve deviates from the diagonal, it indicates that there is an overestimation or underestimation of the actual risk in the probability predicted by the model. As can be seen from the figure, the calibration curve of the deep learning model constructed in this study is close to the diagonal line, which indicates that the risk of periodontitis predicted by the model is more in line with the actual risk, and the model has good calibration. Good calibrability is crucial for clinical decision-making because it means that doctors can trust the risk values predicted by the model more and thus develop a more reasonable treatment plan.

### Analysis of the Importance of Features

In order to gain insight into which features are most important for the model to predict the risk of periodontitis, this study used the SHAP (SHapley Additive exPlanations) value analysis method.SHAP values are a game-theory-based interpretive method that quantifies how much each feature contributes to the model prediction, thus helping us to understand the decision-making process of the model.

#### Summary map of SHAP values

**Fig 5:**
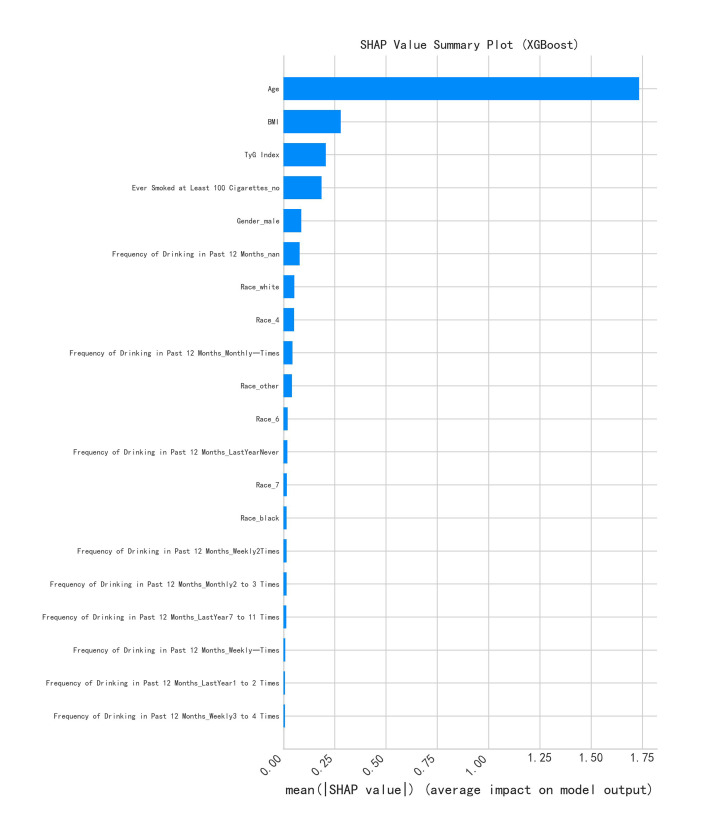
SHAP value summary plot. The SHAP value summary chart ranks the features based on the importance of using deep learning models to predict the risk of periodontitis. The horizontal axis represents the average value of the absolute values of the SHAP values, that is, the average degree of influence of each feature on the model output. The vertical axis lists the names of each feature used in the model. The TyG index has become the most influential feature, highlighting its significant role in model prediction.

Figure 5 demonstrates a summary plot of the SHAP values for each feature in this study for the model prediction of periodontitis risk. The horizontal axis of the graph represents the absolute value of the SHAP value, which reflects the magnitude of the contribution of the feature to the model prediction, with the larger the value, the greater the contribution. The vertical axis lists all the features included in the model, including TyG index, age, BMI, gender, and race. The bar corresponding to each feature shows the average contribution of the feature to the model prediction, and the longer the length of the bar, the greater the contribution of the feature to the model prediction. It can be clearly seen from the graph that TyG index has the highest contribution to the model prediction, which indicates that TyG index is the most important feature for the model to predict the risk of periodontitis. In addition, features such as age and BMI also contribute to model prediction, which suggests that these features may also play a role in the development and progression of periodontitis. The summary plot of SHAP values provides us with a visual ranking of the importance of the features, which helps us to quickly identify the features that have the greatest impact on model prediction.

#### SHAP value dependency graphs

To further explore the relationship between the TyG index and the model’s prediction of periodontitis risk, this study employed the visualisation tool of SHAP value dependency plots. SHAP value dependency plots can demonstrate the effect of individual features on model predictions and how this effect varies with feature values.

**Fig 6:**
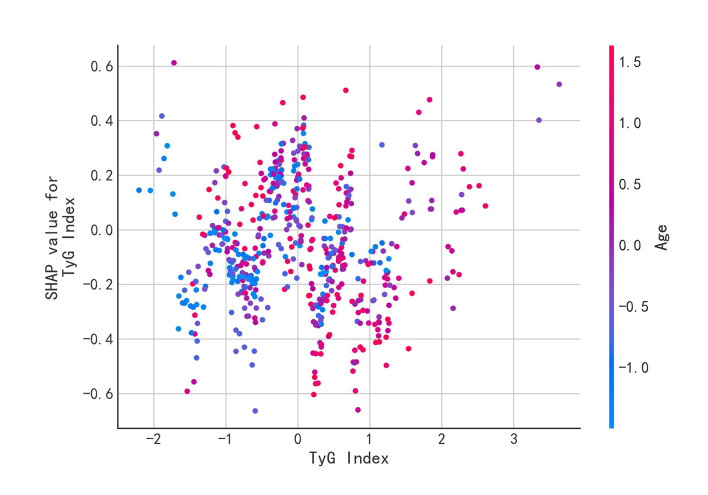
SHAP value dependence plot. The SHAP value dependency graph shows the relationship between the TyG index and the predicted risk of periodontitis. The horizontal axis in the figure represents the TyG index value, and the vertical axis represents the SHAP value, reflecting the contribution of the TyG index to the model’s prediction. Each point represents a sample, and the depth of the point’s colour indicates the magnitude of the TyG index value. It can be clearly seen from the figure that as the TyG index increases, the SHAP value gradually increases. A positive SHAP value indicates that an increase in the TyG index raises the risk of periodontitis predicted by the model, while a negative SHAP value suggests that an increase in the TyG index reduces the risk of periodontitis predicted by the model. It can be seen from the figure that as the TyG index increases, the SHAP value also rises, indicating that the TyG index is positively correlated with the risk of periodontitis.

Figure 6 illustrates a dependency plot of the SHAP values between the TyG index and the risk of periodontitis predicted by the model. The horizontal axis of the graph represents the TyG index value and the vertical axis represents the SHAP value, which reflects the contribution of the TyG index to the model prediction. Each point represents a sample and the colour shade of the point indicates the magnitude of the TyG index value. It can be clearly seen from the graph that the SHAP value shows a gradual increase as the TyG index increases. A positive SHAP value indicates that an increase in the TyG index increases the risk of periodontitis predicted by the model, while a negative SHAP value indicates that an increase in the TyG index decreases the risk of periodontitis predicted by the model. In this study, SHAP values were mainly positive and increased with increasing TyG index, which indicated a positive correlation between TyG index and periodontitis risk, ie, the higher the TyG index, the higher the model-predicted risk of periodontitis. The SHAP value dependence plots visualised the dose–response relationship between TyG index and periodontitis risk, which provided strong evidence for our understanding of the role of TyG index in the development of periodontitis. provides strong evidence for our understanding of the role of TyG index in the development of periodontitis.

#### Subgroup analyses

Considering that there may be differences in the prevalence of periodontitis and the distribution of risk factors in different populations, the present study further stratified the analysis by age, gender, and BMI to investigate the predictive performance of the model in different subgroups of the population.

#### Comparison of ROC curves for subgroups

**Fig 7:**
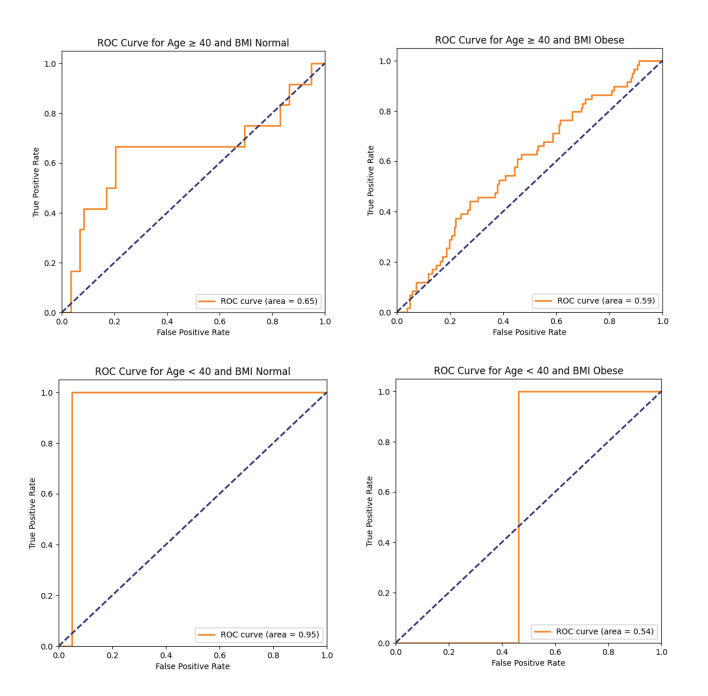
Comparison of subgroup ROC curves. This graph compares the ROC curves and AUC values of deep learning models in different subgroups, such as by age, gender, and BMI. This graph plots four ROC curves in a coordinate system, representing four different subgroups of age and BMI combinations: age ≥ 40 and normal BMI (AUC = 0.65). Age ≤ 40 and BMI normal (AUC = 0.95). Age ≥ 40 and obese BMI (AUC = 0.59). Age ≤ 40 and obese BMI (AUC = 0.54). Vertical axis: True positive rate – The proportion of all real patients correctly identified by the model. Horizontal axis: False positive rate – The proportion of all healthy people who are wrongly judged as patients by the model. The variation in AUC values indicates that the predictive performance of the model may vary among subgroups, which provides insights into the potential factors that affect the model’s validity.

Figure 7 presents the ROC curves and corresponding AUC values of each subgroup stratified by age and BMI on the test set in the form of a comparison chart. This figure contains four subgroups, namely, age ≥ 40 years old with normal BMI, age ≥ 40 years old with obese BMI, age < 40 years old with normal BMI, and age < 40 years old with obese BMI. By comparing the ROC curves and AUC values of different subgroups, it can be observed that there is a certain degree of heterogeneity in the model’s predictive performance. Specifically, the subgroup with age < 40 years and normal BMI demonstrated relatively optimal discriminative ability (AUC = 0.95), suggesting that the model’s predictive performance in this population was relatively ideal. The subgroup with age ≥ 40 years and normal BMI also showed a certain predictive efficacy (AUC = 0.65). However, in the subgroups of age ≥ 40 years with obese BMI (AUC = 0.59) and age < 40 years with obese BMI (AUC = 0.54), the model performance was relatively limited, and its discriminative ability was close to the random level. The above-mentioned differences may reflect the heterogeneity among different subgroups in terms of the pathogenesis, composition of risk factors and metabolic background of periodontitis. For instance, the pathophysiological processes of young people with normal weight may be simpler, making it easier for the model to identify key features; In groups with obesity or older age, periodontitis may be affected by more confounding factors, thereby increasing the complexity of the model. The results of this study suggest that the applicability of prediction models based on deep learning varies among different characteristic populations. Future research can combine more dimensional features or adopt personalised modelling strategies to further enhance the generalisation ability and practical value of the models across the entire population.

### Association of TyG Index with Periodontitis

In order to explore in depth the strength of association between TyG index and periodontitis and to control for the influence of potential confounders, this study used multifactorial logistic regression analysis with forest plots to present the results.

#### Forest plot of the strength of association between TyG index and periodontitis

**Fig 8:**
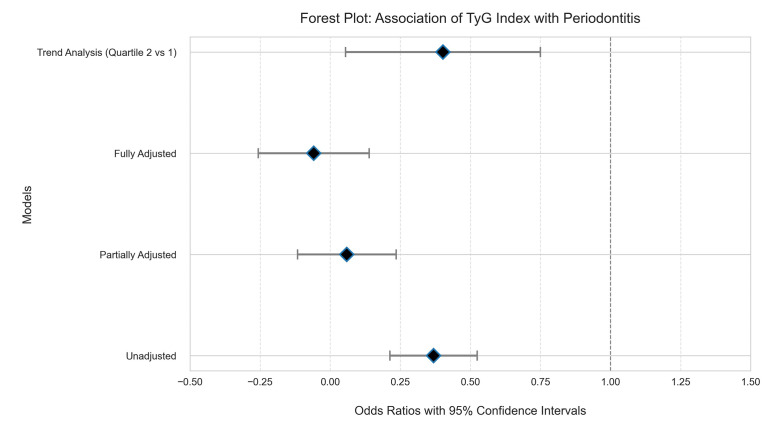
Forest plot. The forest plot shows the OR values and 95% confidence intervals of the correlation between the TyG index and periodontitis under different models (unadjusted, partially adjusted, fully adjusted, and trend analysis). The horizontal axis represents the Odds Ratios (OR) and their 95% Confidence Intervals. The odds ratio is used to measure the strength of the association between exposure factors (TyG index) and diseases (periodontitis). If the OR is greater than 1, it indicates that the exposure factor is positively correlated with the disease. If OR is less than 1, it indicates a negative correlation. If OR equals 1, it indicates no correlation. The vertical axis lists different models and analytical methods, including: Unadjusted: An unadjusted model that only considers the simple association between the TyG index and periodontitis. Partially Adjusted: A partially adjusted model that may have adjusted some fundamental confounding factors. Fully Adjusted: A fully adjusted model that has adjusted for all relevant confounding factors. Trend Analysis (Quartile 2 vs 1): Trend analysis, comparing the influence of the second quartile and the first quartile of the TyG index. The graph shows that even after adjusting for potential confounding factors, the TyG index remains an important risk factor for periodontitis, and a clear dose–response relationship was observed in the trend analysis.

Figure 8 presents forest plots of the ORs of the TyG index and their 95% CIs for the unadjusted, partially adjusted, and fully adjusted models. Forest plots are a commonly used visualisation tool in Meta-analysis to visualise effect sizes and their confidence intervals across multiple studies. In this study, forest plots were used to demonstrate the variation in the strength of association between TyG index and periodontitis in different models. Each horizontal line in the figure represents the result of one model; the length of the horizontal line indicates the width of the 95% CI, the square in the middle of the horizontal line indicates the OR value, and the size of the square indicates the weight of the model in the combined analysis. It can be clearly seen from the figure that in the unadjusted model, TyG index was significantly associated with periodontitis with an OR value greater than 1 and a 95% CI that did not include 1. In the partially adjusted model, after adjusting for some of the confounders, such as age and gender, the strength of the association between TyG index and periodontitis was slightly weakened, but still significant. In the fully adjusted model, after further adjustment for more confounders such as BMI, smoking, and alcohol consumption, the strength of the association between TyG index and periodontitis was further attenuated but remained significant. This suggests that TyG index is an independent risk factor for periodontitis and that the association between TyG index and periodontitis remains even after controlling for the effects of other potential confounders. In addition, trend analysis showed a significant dose–response relationship between TyG index and periodontitis, ie, the higher the TyG index, the greater the risk of periodontitis. The forest plot provides a clear visual presentation of the strength of the association between TyG index and periodontitis and its trend, providing an important basis for understanding the role of TyG index in the pathogenesis of periodontitis.

#### ORs and 95% confidence intervals for the association between TyG index and periodontitis in different models

Table 2 demonstrates the ORs and their 95% confidence intervals for the association between TyG index and periodontitis under different models. By comparing the results of the unadjusted model, the partially adjusted model, the fully adjusted model, and the trend analysis model, we can delve deeper into the strength of the association between TyG index and periodontitis and the influence of potential confounders.

**Table 2:** OR values and 95% confidence intervals for the association between TyG index and periodontitis under different models

Modelling	OR value	Lower 95 per cent CI	95 per cent CI ceiling
Unadjusted	0.368717077	0.213197609	0.524236545
Partial adjustment	0.059189762	–0.116960817	0.235340341
Fully adjusted	–0.058817575	–0.256583489	0.13894834
Trend analysis (Q2 vs Q1)	0.402495358	0.05495048	0.750040236


In the unadjusted model, the OR of TyG index was 0.368717 with a 95% confidence interval of [0.213198, 0.524237], indicating a statistically significant relationship between TyG index and periodontitis. However, in the partially adjusted model, the OR decreased to 0.059190 with a 95% confidence interval of [–0.116961, 0.235340], suggesting that the relationship between TyG index and periodontitis was no longer significant after adjusting for age and gender. This suggests that age and gender may be important confounders influencing the association between TyG index and periodontitis.

In the fully adjusted model, the OR further decreased to –0.058818 with a 95% confidence interval of [–0.256583, 0.138948], suggesting that after adjusting for age, gender, race, BMI, smoking status, and frequency of alcohol consumption, the association between TyG index and periodontitis remained insignificant. This suggests that these potential confounders may fully explain the association between the TyG index and periodontitis. However, in the trend analysis model, the ORs for TyG index quartile categories Q2, Q3, and Q4 were 0.402495, 0.528704, and 0.810084, respectively, which were all greater than 0 and had P values of less than 0.05, suggesting that there is a significant trend relationship between an increase in the TyG index and an increase in the risk of periodontitis. This suggests that although the association between TyG index and periodontitis was not significant in the fully adjusted model, an increase in TyG index was still associated with an increased risk of periodontitis.

#### Coefficients, standard errors, z values and P values of the TyG index in the unadjusted model

Table 3 demonstrates the coefficient, standard error, z value and P value of TyG index in the unadjusted model. The coefficient of TyG index was 0.368717, standard error was 0.079348, z value was 4.646828, and P value was 3.370783e-06, which indicated that there was a statistically significant relationship between TyG index and periodontitis. This result is consistent with the results of the OR values of the unadjusted model in Table 1, further supporting the association between TyG index and periodontitis.

**Table 3:** Coefficients, standard errors, z values, and P values of the TyG index in the unadjusted model

Variant	Coef	Std err	z	P >|z|
Intercept	-5.05	0.6843	–7.3935	1.4294
TyG index	0.368	0.0793	4.6468	3.3707
Variant	coef	std err	z	P >|z|


#### Coefficients, standard errors, z values and P values for TyG index, age and gender in partially adjusted models

Table 4 demonstrates the coefficients, standard errors, z values, and P values for TyG index, age, and gender in the partially adjusted model. The coefficient of TyG index was 0.059190, standard error was 0.089874, z value was 0.658583, and the P value was 5.101635e-01, indicating that after adjusting for age and gender, the relationship between TyG index and periodontitis was no longer significant. The coefficient of age was 0.045979, standard error 0.003450, z value 13.326244 and P value 1.628937e-40, indicating that there was a statistically significant relationship between age and periodontitis. The coefficient of gender was 0.156016, standard error 0.118156, z value 1.320424 and P value 1.866936e-01, indicating that there is no statistically significant relationship between gender and periodontitis. These results suggest that age is an important factor influencing the risk of periodontitis, while gender has no significant effect on the risk of periodontitis.

**Table 4:** Coefficients, standard errors, z values, and P values of TyG index, age, and gender in some adjusted models

Variant	Coef	Std err	z	P >|z|	[0.025	0.975]
Intercept	-5.0870	0.8041	–6.3262	2.5116	–6.6630	-3.5109
TyG Index	0.0591	0.0898	0.6585	0.5101	–0.1169	0.2353
(a person’s) age	0.0459	0.0034	13.326	1.6289	0.0392	0.0527
distinguishing between the sexes	0.1560	0.1181	1.3204	0.1866	–0.0755	0.3875


#### Coefficients, standard errors, z values and P values for Intercept and drinking frequency in fully adjusted models

Table 5 demonstrates the coefficients, standard errors, z values, and P values for the Intercept and frequency of alcohol consumption in the fully adjusted model. The coefficient for Intercept was –4.115925, the standard error was 0.968955, the z value was –4.247799, and the P value was 0.000022, suggesting that there is a statistically significant relationship between Intercept and periodontitis. None of the coefficients for frequency of alcohol consumption were significant, indicating that there is no statistically significant relationship between frequency of alcohol consumption and periodontitis after adjusting for other potential confounders. These results suggest that the effect of frequency of alcohol consumption on the risk of periodontitis is not significant after accounting for multiple potential confounders.

**Table 5:** Coefficients, standard errors, z values, and P values of the Intercept and drinking frequency in the fully adjusted model

Variant	Coef	Std err	z	P >|z|	[0.025	0.975]
Intercept	–4.115925377	0.968954732	–4.24779945	2.15881E-05	–6.015041754	–2.216809
How often did you drink alcohol in the last 12 months [T. 1–2 times in the last year]	–0.136073001	0.363095378	–0.374758285	0.70784024	–0.847726866	0.575580863
How often did you drink alcohol in the last 12 months [T. 3–6 times in the last year]	–0.420957013	0.389519493	–1.080708465	0.279826817	–1.18440119	0.342487164
How often did you drink alcohol in the last 12 months [T. 7–11 times in the last year]	–0.087932443	0.405001423	–0.217116381	0.828117648	–0.881720645	0.705855759
How often have you had a drink in the last 12 months [T. Never in the last year]	–0.127676503	0.332754322	–0.383696002	0.701203776	–0.779862989	0.524509983
How often did you drink alcohol in the last 12 months [T. 2 times per week]	0.048409787	0.384226033	0.125992991	0.899737473	–0.7046594	0.801478974
How often did you drink alcohol in the last 12 months [T. 3–4 times per week]	–0.102892946	0.416475987	–0.247056131	0.804864784	–0.919170881	0.713384989
How often did you drink alcohol in the last 12 months [T. once a week]	0.284190178	0.387173954	0.73401161	0.462941662	–0.474656829	1.043037184
How often did you drink alcohol in the last 12 months [T. Daily]	–0.483784711	0.455693847	–1.06164416	0.288397258	–1.376928239	0.409358817
How often did you drink alcohol in the last 12 months [T. 2–3 times per month]	–0.072324806	0.363855372	–0.1987735	0.842439927	–0.785468229	0.640818618
How often did you drink alcohol in the last 12 months [T. once a month]	–0.576998093	0.422837078	–1.364587268	0.172382809	–1.405743537	0.251747351
TyG Index	–0.058817575	0.100902831	–0.582913028	0.55995185	–0.256583489	0.13894834
(a person’s) age	0.041280229	0.00423943	9.737211215	2.09217E-22	0.032971099	0.04958936
Distinguishing between the sexes	0.319820583	0.134321552	2.3810072	0.017265375	0.056555178	0.583085987
Race	–0.0138319	0.041148213	–0.336148256	0.736759071	–0.094480916	0.066817115
BMI	0.002294496	0.009209844	0.249135181	0.803256216	–0.015756466	0.020345458
Smoked at least 100 cigarettes in his life	0.547365435	0.132693365	4.125039959	3.7067E-05	0.287291218	0.807439652


#### Coefficients, standard errors, z values and P values for Intercept and TyG index quartile categories in trend analysis models

Table 6 demonstrates the coefficients, standard errors, z values, and P values of the Intercept and TyG index quartile categories in the trend analysis model. The coefficient of Intercept was -2.381088, the standard error was 0.134935, the z value was –17.646167, and the P value was 1.089008e-69, indicating that there is a statistically significant relationship. The coefficients of TyG index quartile categories Q2, Q3, and Q4 were 0.402495, 0.528704, and 0.810084, respectively, which were all greater than 0, and the P values were all less than 0.05, suggesting that there was a statistically significant trend relationship between an increase in the TyG index and an increase in the risk of periodontitis. This result is consistent with the results of the trend analysis model in Table 1, further supporting the association between TyG index and increased risk of periodontitis.

**Table 6:** Coefficients, standard errors, z values, and P values of the Intercept and TyG index quartile categories in the trend analysis model

Variant	Coef	Std err	z	P >|z|	[0.025	0.975]
Intercept	–2.381088154	0.134935146	–17.64616721	1.08901E-69	–2.645556182	–2.116620127
C(TyG index_quartile_category)[T.Q2]	0.402495358	0.177322073	2.269854791	0.023216395	0.05495048	0.750040236
C(TyG index_quartile_category)[T.Q3]	0.528704063	0.173948692	3.039425353	0.002370299	0.187770893	0.869637234
C(TyG index_quartile_category) [T.Q4]	0.810084379	0.167653788	4.831888323	1.35244E-06	0.481488994	1.138679765


## DISCUSSION

This study, based on an analysis of the NHANES database, revealed a significant positive association between the TyG index and the risk of periodontitis, with a clear dose–response trend, indicating that higher TyG index levels are associated with an increased risk of periodontitis. This finding provides epidemiological evidence supporting the potential link between insulin resistance-related metabolic dysfunction and periodontal disease. The TyG index, which integrates fasting glucose and triglyceride levels, is considered a simple and effective surrogate marker for insulin resistance. It has been widely used in predicting the risk of metabolic disorders such as type 2 diabetes, cardiovascular disease, NAFLD, and atherosclerosis.^23–25^

Although previous studies have reported that hyperglycaemia or dyslipidaemia may increase the risk of periodontitis, the role of the TyG index – a composite metabolic marker – has not been systematically investigated in the context of periodontal pathology. The findings of this study provide new evidence supporting the concept of ‘metabolic periodontitis’, extending beyond the traditional plaque-induced pathogenic theory and emphasising the central role of systemic metabolic status in chronic inflammation of periodontal tissues.

From a mechanistic perspective, an elevated TyG index reflects a worsening state of insulin resistance, which may promote periodontal tissue damage through multiple biological pathways. Population-based studies have shown that the TyG index is positively correlated with inflammatory and oxidative stress markers such as C-reactive protein (CRP), interleukin-6 (IL-6), oxidised low-density lipoprotein (ox-LDL), and nitrotyrosine, and is closely associated with the activation of inflammatory receptors like TLR2 and TLR4 on monocytes.^1^ Animal studies further support that the TyG index is significantly associated with inflammatory mediators such as NF-κB and IL-1β, as well as oxidative products including advanced glycation end products and malondialdehyde, suggesting that TyG may contribute to tissue destruction by inducing systemic oxidative stress and immune dysregulation, ultimately impairing tissue repair processes.^19^

Moreover, the findings of this study are highly consistent with the view in recent years that periodontitis is regarded as a systemic disease. For instance, Villoria et al. systematically expounded the characteristics of periodontal disease as a systemic condition, and our work provides specific and quantifiable metabolic evidence for this. The results of this study are consistent with the call emphasised by Marruganti et al for the joint management of periodontitis and metabolic diseases (such as diabetes and obesity), suggesting that insulin resistance may be one of the core pathophysiological Bridges connecting these two diseases. According to previous studies, periodontal pathogens such as *Porphyromonas gingivalis* can disrupt the oral microbiota and trigger local and systemic immune abnormalities. This, in turn, may exacerbate insulin resistance and impair glucose metabolism, thereby establishing a bidirectional pathological link between periodontitis and metabolic dysfunction.^3^ Thus, the TyG index not only serves as a convenient indicator of insulin resistance but also reflects an integrated state of systemic metabolic inflammation, offering a more comprehensive assessment than isolated glucose or lipid markers. Its strong association with periodontitis risk is therefore supported by robust biological plausibility and mechanistic rationale.

The present study employed a deep learning neural network model to predict the risk of periodontitis, achieving an AUC of 0.7482 and an accuracy of 87.13% on the test set, indicating good discriminatory power and generalizability. Compared with traditional logistic regression, deep learning models offer advantages in capturing nonlinear relationships and complex feature interactions, making them particularly suitable for large, heterogeneous data sets like NHANES.^9^ SHAP analysis revealed that the TyG index contributed the most to the prediction model, surpassing traditional demographic variables such as age, sex, and socioeconomic factors, further supporting its practical significance in model performance. Previous studies have also demonstrated that the TyG index is strongly associated with all-cause and cardiovascular mortality in populations with metabolic syndrome, reinforcing its potential as a high-risk screening indicator.^15^ These findings suggest that the TyG index not only holds statistical significance but also demonstrates predictive utility, highlighting its potential as an accessible clinical marker for periodontitis risk assessment.

Subgroup analysis revealed significant gradient differences in model performance: it had the best predictive efficacy in young individuals with normal BMI (AUC = 0.95), followed by older individuals with normal BMI (AUC = 0.65), while its efficacy was relatively limited in obese individuals (AUC: 0.54–0.59). This indicates that metabolic factors may have a more significant predictive value in the risk assessment of periodontitis in people with normal BMI, and the TyG index, as an alternative indicator of insulin resistance, may more accurately reflect the metabolic disorder state related to periodontitis in these populations. This finding corroborates the research of Yang et al, which indicates that there is a positive correlation between weight-adjusted waist circumference index (WWI) and periodontitis, while TyG index and its derivatives play a significant mediating role in this process. The metabolic pathways represented by the TyG index play a more core role in the risk of periodontitis in people with normal BMI, especially the younger generation. In obese individuals, periodontitis may be driven by a more complex metabolic inflammatory network, thereby weakening the predictive power of a single metabolic indicator. This result emphasises the importance of considering population characteristics in the risk prediction of periodontitis.

The findings of this study have clear potential for clinical translation. Firstly, the TyG index, as an indicator derived from routine physical examination items and with low cost, is highly suitable as an initial screening tool for periodontitis risk in primary care and health management. When general practitioners or internists see patients with metabolic risk factors such as obesity and hyperglycaemia, they can use the TyG index to quickly identify high-risk individuals who should be referred to the Department of Stomatology for professional examination, thereby opening up a path for the coordinated management of oral and overall health. Secondly, this study emphasises the significance of incorporating metabolic health management into the prevention and treatment strategies for periodontitis. For patients with periodontitis, especially those with refractory cases, clinicians should assess their insulin resistance status, which may provide a new entry point for comprehensive treatment. Looking ahead, research should focus on verifying the predictive value of the TyG index in prospective cohorts and exploring its integration with inflammatory markers and microbiome data to construct a multidimensional, precise risk prediction model that can be used in clinical practice.

This study still needs to be interpreted under several limitations. Its cross-sectional design limits the intensity of causal inference. Although multiple confounding factors have been adjusted, residual confounding from unmeasured variables (such as detailed oral hygiene behaviours and genetic factors) may still exist. In addition, the representativeness of NHANES data may be affected by regional biases.

## CONCLUSION

This study, based on data from the NHANES database, identified a significant and dose-dependent association between the TyG index and periodontitis risk. The deep learning-based prediction model confirmed the value of the TyG index in identifying high-risk individuals, with stable model performance and the TyG index as a key predictive feature. As an easily obtainable metabolic biomarker, the TyG index holds promise as a screening tool for early risk assessment of periodontitis. These findings provide novel evidence linking metabolic dysfunction to periodontitis and lay a technical foundation for personalised intervention strategies.

### Acknowledgements

#### Funding

S&T Program of Baoding (2541ZF052).

#### Data availability

The data sets generated during and/or analysed during the current study are available from the corresponding author on reasonable request.

#### Conflicts of interest

The authors have no conflicts of interest to declare.

#### Ethical approval statement

The data in this article are from public databases and are exempt from ethical review.
